# Clinical effect of vitamin C supplementation on bone healing: A systematic review

**DOI:** 10.4317/medoral.24944

**Published:** 2022-04-03

**Authors:** Kevin Barrios-Garay, Jorge Toledano-Serrabona, Cosme Gay-Escoda, Mª Ángeles Sánchez-Garcés

**Affiliations:** 1 DDS. Private practice; 2 DDS. Fellow of the Master’s degree programme in Oral Surgery and Implantology, Faculty of Medicine and Health Sciences, University of Barcelona. Researcher at IDIBELL (Bellvitge Biomedical Research Institute), Barcelona, Spain; 3 MD, DDS, MS, PhD, EBOS, OMFS. Chairman and Professor of Oral and Maxillofacial Surgery, Faculty of Medicine and Health Sciences, University of Barcelona. Director of the Master Degree Program in Oral Surgery and Implantology (EHFRE International University/FUCSO). Coordinator/Researcher at the IDIBELL institute. Head of the Department of Oral Surgery, Implantology and Maxillofacial Surgery, Teknon Medical Center, Barcelona, Spain; 4 MD, DDS, MS, PhD, EBOS. Lecturer in Oral Surgery. Master’s degree program in Oral Surgery and Implantology, Faculty of Medicine and Health Sciences, University of Barcelona. Researcher at IDIBELL (Bellvitge Biomedical Research Institute), Barcelona, Spain

## Abstract

**Background:**

The aim of the present systematic review was to evaluate the clinical effect of vitamin C on bone healing after bone fracture or bone reconstruction procedures.

**Material and Methods:**

In October 2020, Cochrane Library, Scopus and PubMed-Medline databases were searched without restrictions to identify animal and human studies that fulfilled the eligibility criteria. Outcome measures were bone healing time, bone gain (mm), bone density and adverse events. The risk of bias assessment of the selected studies was evaluated by means of Cochrane Collaboration’s Tool for randomized clinical trials, while randomized clinical animal trials were assessed according to SYRCLE’s tool. Additionally, quality of reporting animal studies were assessed according to ARRIVE guidelines.

**Results:**

Out of the 248 articles that yielded the initial search, 11 papers about the effect of ascorbic acid on bone healing were selected. In most of the animal studies, vitamin C seemed to accelerate bone formation owing to an enhanced osteoblastic proliferation and differentiation and its antioxidant function when pro-oxidant substances were added. It was not possible to observe this phenomenon in human studies.

**Conclusions:**

Although additional well-performed animal and human studies are required, vitamin C seems to accelerate bone regeneration without adverse events. However, it is not possible to recommend a specific dose or route of administration of vitamin C to improve the bone healing process in humans as there was great heterogeneity among the included studies.

** Key words:**Vitamin C, Fracture healing, Bone regeneration, Bone mineral density, Implants.

## Introduction

Vitamin C (vit C) or ascorbic acid (chemical name: 2,3-didehydro-L-threo-hexane-1,4-lactone) is a water-soluble vitamin obtained from natural or synthetic sources that plays an important role in many biological reactions (1). This vitamin is synthesized from glucose in the liver of most mammalian species, but not in humans or other animal groups (non-human primates, guinea pigs and bats) (2).

An imbalance between the production of reactive oxygen species (free radicals) and antioxidant substances can lead to cell damage and be the cause of various conditions (2). However, vitamin C, as an electron donor, can eliminate hydroxyl and superoxide radicals and, therefore, prevent cell damage by protecting the capillary endothelium and circulating cells (2-4). On the other hand, the importance of vitamin C in bone metabolism is also remarkable, since it is related to the hydroxylation of collagen (5-7), and to the expression of no-collagenic proteins such as alkaline phosphatase, osteonectin and osteocalcin (8). Besides, vit C promotes the expression of genes related to chondrocytes differentiation and is involved in osteoblastogenesis and osteoclastogenesis (4). Indeed, Urban *et al*. (9) showed that the addition of vit C in concentrations up to 200 g/ml in cell cultures had a positive effect on osteoblast proliferation and also increased type-I collagen synthesis.

Severe vitamin C deficiency results in scurvy, a disease that is characterized by weakening of collagenous structures, resulting in poor wound healing and impaired immunity (10). Currently, it is difficult to find this condition among the population, however, there are some groups that have a higher requirement of vit C, such as the elderly, alcoholics, smokers and diabetics (10,11). In addition, preclinical and clinical studies have shown that vitamin C deficiency causes a delay in tissue healing and inhibits collagen synthesis (1,12). Furthermore, this deficiency has been linked to an increased risk of osteoporosis and fractures due to decreased bone formation (4).

Bone defects can heal totally or partially depending on local or systemic factors (13,14). When spontaneous bone regeneration is not achieved, additional measures are needed, such as specific surgical techniques and materials (13,15). Several articles have shown that vit C can improve bone healing during regeneration procedures, however, this topic has not yet been systematically reviewed. Thus, a systematic review of animal and human studies investigating the efficacy of the use of vit C as a supplement to a bone healing procedure may add new information.

The purpose of this study was to evaluate the current knowledge on the efficacy of vit C in bone regeneration, as well as to stablish a protocol of dosage and posology of vit C to improve bone healing process.

## Material and Methods

The present systematic review was performed according to the statements of “Preferred Reporting Items for Systematic Reviews and Meta-Analyses” (PRISMA) (16).

- Eligibility criteria

The focus questions to be addressed were:

How can systemic or oral administration of vit C influence the bone healing process in terms of speed and quality? Are there any differences compare to the bone healing process without Vit C administration? Accordingly, articles that fulfilled the following eligibility criteria were selected (PICO parameters):

(P) Population: Patients or animals treated with vit C.

(I) Intervention: Bone healing using systemic or oral administration of vit C.

(C) Comparison: Bone healing without the use systemic or oral administration of vit C.

(O) Outcomes: For both animal and human studies bone healing time, histomorphometry (bone gain (mm) bone density (HU)) and number of adverse effects were assessed. Additionally, gene/cytokines expression was only assessed in animal studies.

Inclusion criteria were human and animal studies that evaluated the effect of systemically or oral administered vitamin C in terms of bone healing. Studies that evaluated the effect of different vitamins simultaneously were excluded. No restrictions were applied regarding the language and the year of publication.

- Information sources and search strategy

A systematic search in the Cochrane Library, Scopus and PubMed-MEDLINE databases was conducted in October 2020. The following search strategy was used:

1) PubMed-MEDLINE: ("ascorbic acid" [MH] OR "acid, ascorbic" [TIAB] OR "L-ascorbic acid" [TIAB] OR "acid, L-ascorbic" [TIAB] OR "vitamin C" [TIAB]) AND ("bone regeneration" [MH] OR "osteoconduction" [TIAB] OR "bone transplantation" [MH] OR "bone grafting" [TIAB] OR "guided tissue regeneration" [MH] OR "bone remodeling" [MH] OR "fracture healing" [MH] OR "osseointegration" [MH]).

2) Scopus: TITLE-ABS-KEY((“ascorbic acid" OR "vitamin C") AND ("bone regeneration" OR "fracture healing" OR "osseointegration")).

3) Cochrane Library: (“ascorbic acid" OR "vitamin C") AND ("bone regeneration" OR "fracture healing" OR "osseointegration").

Additionally, a cluster search and a manual search of articles published during the last 10 years in "Journal of Clinical Periodontology", "Journal of Periodontal Research", "Clinical Oral investigations", "Journal of Oral and Maxillofacial Surgery" "Medicina Oral Patología Oral y Cirugía Bucal", "Oral Surgery Oral Medicine Oral Pathology Oral Radiology", "Journal of Dentistry", "The International Journal of Oral and Maxillofacial Implants" and "Clinical Oral Implants Research" were carried out. Grey literature was also explored through the Bielefeld Academic Search Engine (BASE).

- Selection process of studies

The selection of the studies was made by two independent reviewers (K.B-G. and J.T-S.). After removing duplicates and screening the remaining articles reading by their title and by their abstract, the studies that fulfilled the eligibility criteria were selected. A third reviewer (M.Á.S-G) with broad experience in systematic reviews resolved any disagreement during the article selection process. Cohen’s kappa was calculated to measure the level of agreement between the two reviewers.

- Data collection process and synthesis of the results

A qualitative synthesis was performed using data extraction Tables. The following information was retrieved from the selected articles: name of the authors, year of publication, study design, number of participants, description of experimental groups, type of bone defect, vitamin C dosage, exposition route and administration frequency, follow-up time and outcomes variables (healing time, bone gain measured in mm, adverse effects). If necessary, authors of the selected studies were contacted for clarification missing or incomplete data.

Since high heterogeneity was found among the selected studies, a quantitative synthesis was not carried out.

- Risk of bias and quality assessment of the included studies

Risk of bias and quality assessment of the included studies was conducted by two independent reviewers (K.B-G and J.T-S). A third reviewer (M.Á.S-G) resolved any disagreements.

Randomized clinical trials (RCT) were evaluated by means of “Cochrane Handbook for Systematic Reviews of Interventions” (17). The following items were classified in low, unclear or high risk of bias: random sequence generation, allocation concealment, patient blinding, outcome blinding, incomplete outcome data and selective reporting. Additionally, the “SYRCLE tool for assessing the risk of bias of animal intervention studies” was used to assess the risk of bias of randomized clinical animal trial (RCAT) studies (18). The following items were classified in low, unclear or high risk of bias: sequence generation, baseline characteristics, allocation concealment, random housing, blinding of the intervention to caregivers and researchers, random outcome assessment, blinding of outcome assessor, incomplete outcome data, selective outcome reporting and other sources of bias. On the other hand, quality of reporting animal studies were assessed according to ARRIVE guidelines (19) for *in vivo* experiments and assigned predefined grades (study design, sample size, inclusion and exclusion criteria, randomization, blinding, outcome measures, statistical methods, experimental animals, experimental procedures and results). Any disagreement during this step was resolved thanks to one independent investigator (M.Á.S-G).

## Results

- Study selection

The initial search yielded 253 studies after eliminating duplicates. After discarding 210 studies by reading their title and 33 by reading the abstract. the full-text of 12 articles was assessed for elegibility (1,11,20-29). Only one study was excluded because evaluated the effect of calcium ascorbate supplemented with vit C metabolites (21). Finally, 11 studies, written in English, were included in the present systematic review; nine animal studies (1,22-29), and two randomized clinical trials (11,20). The level of agreement between the two reviewers was 93.75% with a Cohen’s kappa statistic of 0,84.

**Figure 1:**
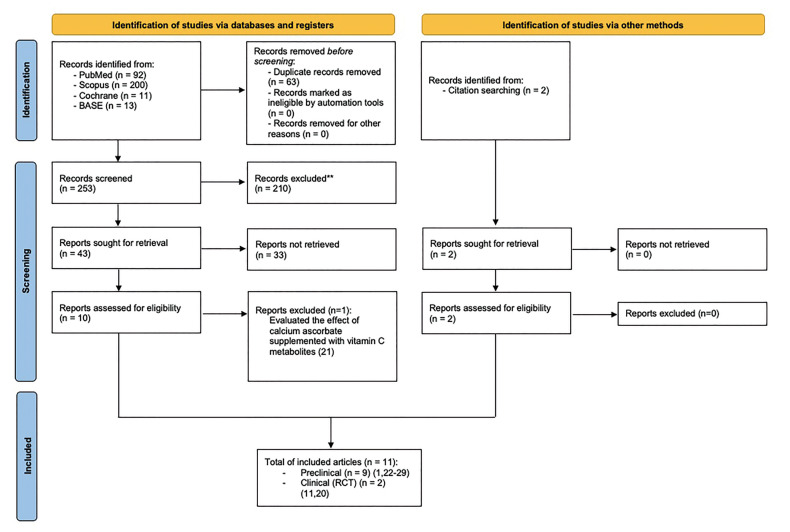
Flow-chart of the review process following PRISMA statements.

Fig. 1 shows the flow-chart of the study selection process.

- Risk of bias and quality assessment of the included studies

**Figure 2:**
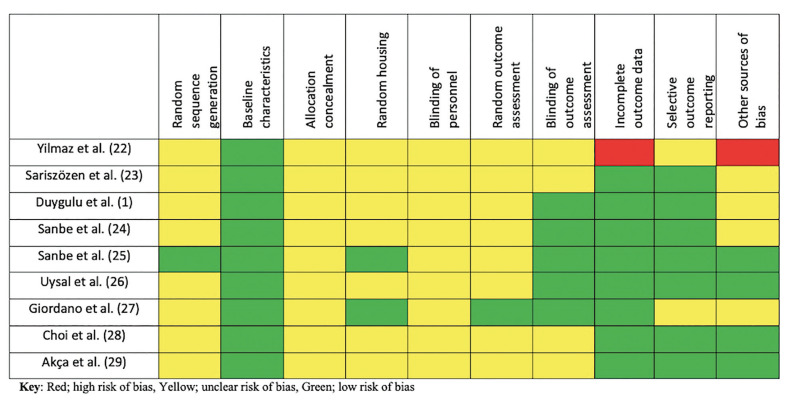
Assessment of quality and risk of bias of included animal studies.

Regarding animal studies, one study has a high risk of bias and low quality of reporting (22) because it has attrition bias, another has an unclear risk of bias and low quality of reporting (27), other two studies have an unclear risk of bias and an unclear quality of reporting (23,28) and five studies have an unclear risk of bias but high quality of reporting (1,24-26,29) (Fig. 2).

**Figure 3:**
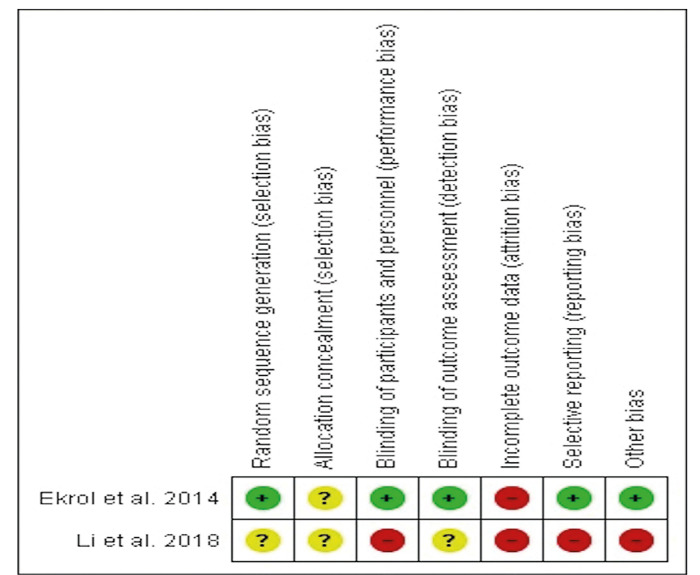
The Cochrane collaboration’s tool for assessing risk of bias for randomized.

Regarding human studies, both were classified as having high risk of bias. The study of Ekrol *et al*. (11) because it had an attrition bias, whereas the study of Li *et al*. (20) had a high risk of bias due mainly to performance, attrition and reporting bias (Fig. 3).

- Qualitative synthesis

None of the included studies assessed bone gain or bone quality outcomes.

Animal studies

The nine animal studies included comprised 334 rats over a follow-up period ranging from 20 to 98 days. All the studies evaluated the effect of vitamin C in the following clinical scenarios: on bone healing after tibial fracture (22,23,27), on bone defects healing with the addition of pro-oxidant substances (zymosan and nicotine) (1,29), on alveolar bone resorption in rodents fed cholesterol-rich diets (24,25), in bone formation during the expansion of the inter-premaxillary suture (26) and in osteogenic differentiation and osteoclast formation in ovariectomized rats (a useful animal model for evaluating the effect of osteoporotic treatments on the skeletal system) (28) (Table 1).

In Table 2 is depicted the main results of the included animal studies. Two of them (22,23) showed that vit C administration accelerated bone healing after fracture when administered systemically at doses ranging from 0,5 mg/kg to 200 mg/kg. This effect was also observed when pro-oxidant substances were added (1), but a daily dose of 500 mg/kg was necessary. In fact, Akça *et al*. (29) found that vit C reversed the negative effects produced by nicotine administration on bone healing. In this line, two papers from the same group (24,25) found that vit C diluted in water at concentrations of 1g/l and 2g/l reversed the negative effects on alveolar bone produced by a cholesterol-rich diet (decreased bone density and osteocalcin levels higher expression of hexanoyl-lysine, 8-hydroxideoxyguanosin, nuclear factor kappa beta and RANKL). Conversely, the study by Giordano *et al*. (27) did not show any benefit from vit C administration at a dose of 200 mg/kg on bone healing after tibial fracture.

Regarding the route of administration, Uysal *et al*. (26) observed that during inter-premaxillary suture expansion, the systemic administration of vit C had significantly better results than local administration of vit C, both at a dose of 0.5 mg/kg. On the other hand, Choi *et al*. (28) observed that oral administration of vit C at doses of 3 mg/kg, 7.5 mg/kg and 15 mg/kg improved bone mineral density and increased the expression of genes involved with osteoblastic differentiation (BMP-2, SMAD 1/5/8, RNTF-2, osteocalcin and COL1) and decreased the expression of genes related to osteoclastic differentiation (RANK, RANKL, TRAP and cathepsin-K) in ovariectomized rats.

Human studies

Regarding the human studies, two randomized clinical trials met the inclusion criteria. These studies comprised 464 patients during a follow-up period ranging from 14 to 365 days (Table 3).

In Table 4 is depicted the main results of the included human studies. Ekrol *et al*. (11) studied the effect of vit C by oral supplementation at daily dose of 500 mg/day for 50 days in 336 patients with displaced and non-displaced distal radial fractures, of whom 87 (25,9%) dropped out within the follow-up period. No statistically differences in , terms of bone healing were observed (11). On the other hand, the study by Li *et al*. (20) evaluated the effect of oral administration of vit C for 7 days at dose a 300 mg/day after dental implant placement in different clinical scenarios in 128 patients (no patient dropped out). After 14 weeks of follow-up, they observed that vit C improved soft tissue healing (*P* < 0.05) after dental implant placement in patients who undergoing guided bone regeneration procedures. However, there were no benefits after vit C supplementation in terms of pain management. - Bone healing or implant success were not assessed in the present study.

Since high heterogeneity was found among the selected studies, a quantitative synthesis was not carried out.

**Table 1:**
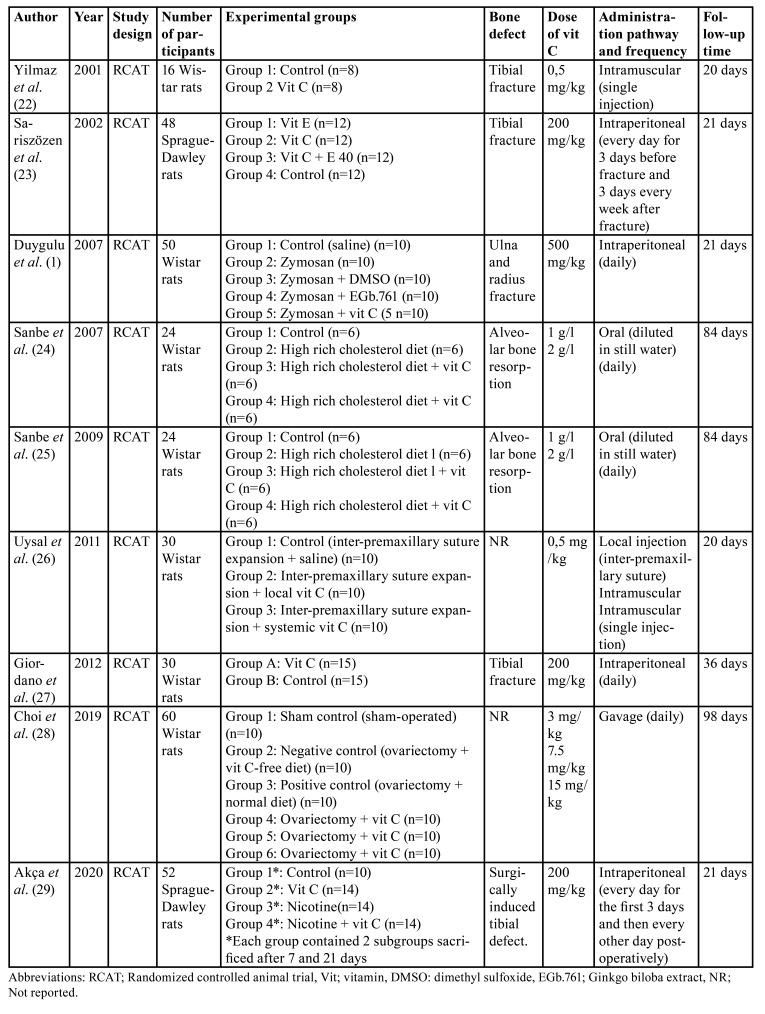
Description of the included animal studies.

**Table 2:**
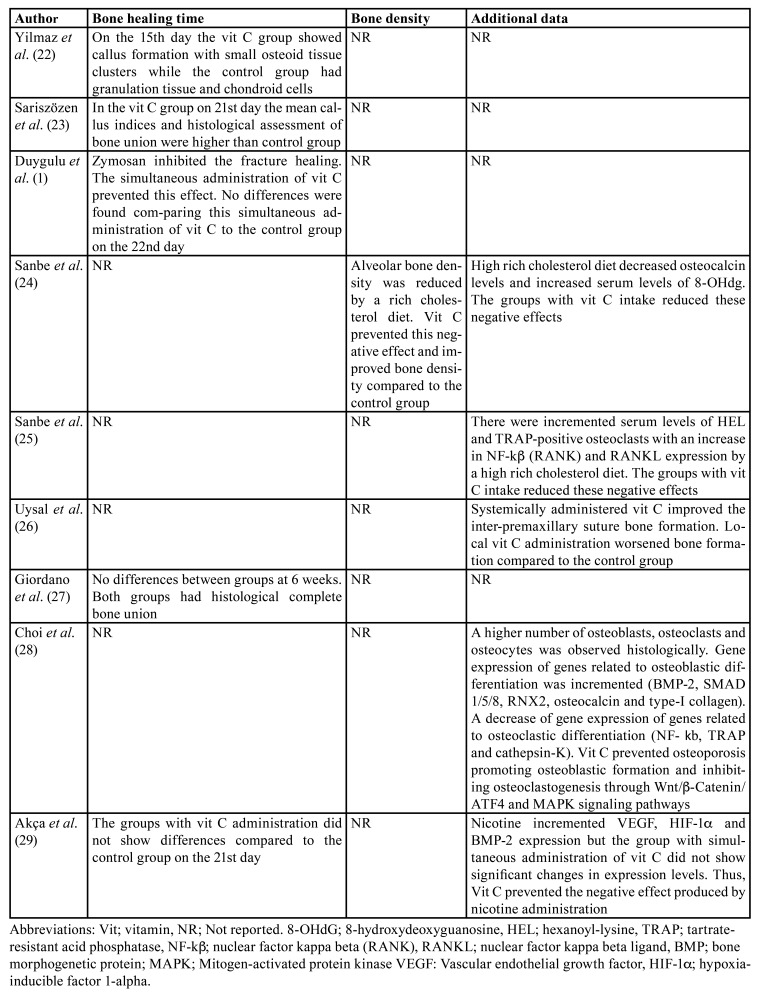
Outcomes of the included animal studies.

**Table 3:**
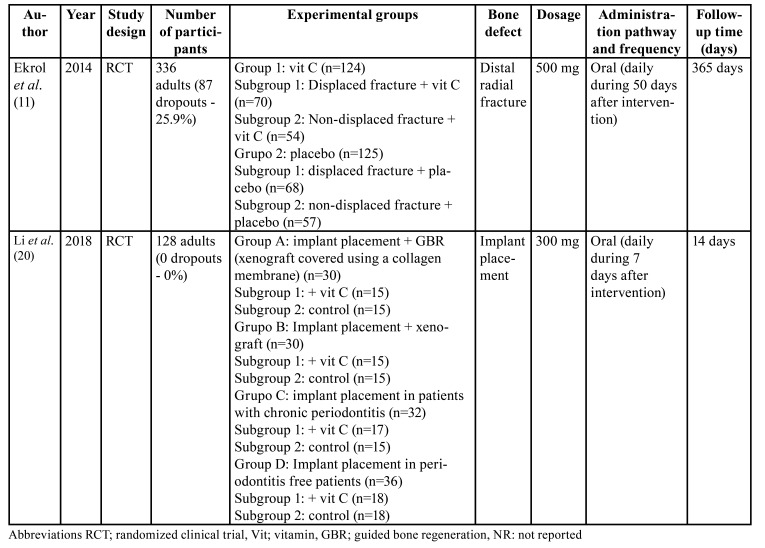
Description of the included human studies

**Table 4:**
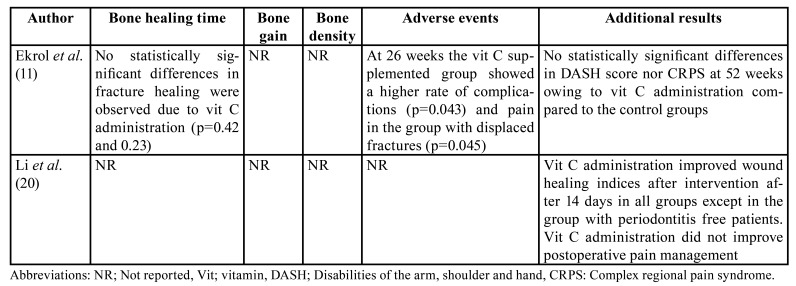
Outcomes of the included human studies.

## Discussion

According to the results of the included animal studies, the administration of vit C seems to improve bone healing and bone formation, as vit C may modulate osteoblastogenesis and osteoclastogenesis, and also has an antioxidant function. However, data extracted from the included RCTs did not show any additional benefits of oral vit C supplementation on either bone healing or bone regeneration.

Different results were found regarding bone healing speed among the included animal studies. This fact could be due to the ability of rodents to synthesize vit C from a normal diet (30). This makes it necessary to know exactly the dietary pattern of the animals. Thus, whereas the rodents in the study by Giordano *et al*. (27) had unlimited access to food, the feeding pattern followed in the studies by Yilmaz *et al*. (22) and Sariszözen *et al*. (23) was not exactly explained.

Uysal *et al*. (26) described that locally injected vit C had a negative impact on bone formation after expanding the inter-premaxillary suture. The authors explained that this could be due to an alteration in apoptotic regulation related to bone healing (26). However, the lack of further studies evaluating the local effect of vit C makes it impossible to know if this is an isolated phenomenon or if it is really due to this route of administration. Nevertheless, the authors observed that systemic administration of vit C obtained good results in terms histomorphometry of bone formation (26).

A minimum intake of 75 mg/day for adult women and 90 mg/day for adult men of vit C has been recommended in The United States of America and Canada (4). Additionally, an increase of this intake values is recommended in 15 mg/day for pregnant women, 50 mg/day if lactating, and 35 mg/day for smokers (4).

Smokers are constantly exposed to a source of prooxidant substances and reactive oxygen species that lead to an increased requirement of vit C (3). However, taking into account that nicotine also has a negative effect on osteoblastic proliferation (31) and that the vit C requirement is higher in smokers (2,4), we believe that vit C supplementation should be indicated in these patients, especially when they undergo bone regeneration procedures of the jaws or after traumatic injuries. In our review, two studies (1,29) induced the formation of free radicals and reactive oxygen species by administration of zymosan and nicotine. The authors observed that vit C acts as an antioxidant by scavenging these free radicals (1-3), which explains the good bone healing reported in these studies. In this line, Tomofuji*et al*. (32) evaluated Wistar rats fed a cholesterol-rich diet and demonstrated that this type of diet can initiate and increase bone loss around the teeth.However, it seems that, as demonstrated in the studies of Sanbe*et al*. (24,25), vit C prevents the negative effects produced by this type of diet.This is mainly due to the inhibition of lipid peroxidation and an increase in osteoblastic proliferation and differentiation genes, as well as a decrease in osteoclastic prolifer- ation and differentiation genes.

Regarding human studies, Ekrol *et al*. (11) did not observe an improved bone healing due to vit C administration, however, they pointed out that the benefits of vit C administration may only be observable in vit C-deficient populations On the other hand, Li *et al*. (20) suggested that vit C improves surgical wound healing after dental implant placement, but the study did not evaluate the effect of vit C on bone regeneration by radiography or histology.

The scientific literature describes some adverse effects associated with vit C supplementation, such as diarrhea and abdominal pain, with high dose in a single administration. Hyperuricosuria has been described in vit C concentrations higher than 3 g, hyperoxaluria in concentrations higher than 1 g, hyperoxalemia in patients treated with hemodialysis when administered intravenously repeatedly in doses of 1 g and hemolysis in patients with phosphate-6-glucose dehydrogenase deficiency administered intravenously or orally when the concentration is higher than 6 g in a single dose (33). In our review, none of the selected studies reported any of the adverse events mentioned above.

Regarding the use of other vitamins to aid bone formation and healing, vitamins D and E have also been studied. Carinci *et al*. (34) studied vitamins C and E to evaluate their effect on preosteoblast gene expression. Vitamin E showed no effect, whereas vitamin C modified preosteoblast genes by increasing cell growth, metabolism, morphogenesis and cell communication. Similarly, one of the studies included in this review used vit E on bone healing (23), and showed no additional benefit, not even associated with vit C. On the other hand, the use of vit D supplementation can be useful in patients with osteoporosis as vit D has a crucial role on bone mineralization. In fact, vit D deficit has been associated to a worse dental implant osteointegration and an increased risk of early implant failure (35). However, as with vit C, further studies are still required to confirm the clinical effect of their oral supplementation.

Finally, there are several limitations related to the present systematic review that should be mentioned. First, most of the included studies were animal studies which may represent a problem in the external validation of their results. Furthermore, only two RCTs with low quality related to vit C administration could be included. Another limitation was the heterogeneity found among the selected articles, which prevents us from comparing the results obtained with respect to bone defect, different bone regeneration techniques and populations with higher vit C requirements (elderly, diabetics and smokers). To overcome these limitations, it is necessary to perform well-designed RCTs to determine whether vit C supplementation in standardized models of bone defects implies any benefit in the speed of healing or in the quality of bone obtained in patients without dietary vit C deficiency. An experimental animal model with a cranial bone defect of critical size could be useful to compare various groups (test and control), in terms of histomorphometry, at different time points and vitamin C doses. The defect proposed by Higuchy *et al*. (36) or by Kustro *et al*.(37) or especially by Liu *et al*. (38) because of its dimension, will help to confirm the null hypothesis of the present work.

There is enough evidence that vitamin C has good results at the *in vitro* level regarding osteoblast differentiation and maturation and, it is possible to think that it could be a very easy way to improve bone and soft tissue healing conditions, without increasing morbidity and cost of a treatment.

Bone tissue regeneration and dental implant placement are increasing, especially among elderly patients. If our hypothesis is confirmed, recommending vit C supplementation during the bone healing period could be an effective, inexpensive and easy-to-implement treatment. These supplements could be especially useful in smoking patients, and hypercholesterolemic patients due to the requirement of antioxidants.

The techniques used in oral and implant surgery are constantly improving using different materials, some of them experimental or expensive, and it seems that there is no room for simplicity and, in certain cases, less could be more.

## Conclusions

Although additional well-performed animal and human studies are required, vitamin C seems to accelerate bone regeneration without adverse events. However, it is not possible to recommend a specific dose or route of administration of vitamin C to improve the bone healing process in humans as there was great heterogeneity among the included studies.
